# Neuroprotective Role of AQP4 Knockdown in Astrocytes After Oxygen–Glucose Deprivation

**DOI:** 10.1002/brb3.70107

**Published:** 2024-10-23

**Authors:** Xin Xing, Shuyan Zhang

**Affiliations:** ^1^ Department of Neurology The Fourth Affiliated Hospital of Harbin Medical University Harbin Heilongjiang China

**Keywords:** astrocytes, AQP4, MCAO, neuroinflammation

## Abstract

**Background:**

Aquaporin‐4 (AQP4), predominantly expressed in astrocytes, has been implicated in the development of brain edema following ischemic events. However, its role in post‐stroke neuroinflammation is not fully understood.

**Methods:**

Using a middle cerebral artery occlusion (MCAO) mouse model, we assessed AQP4's role in post‐stroke inflammation. Brain tissue slices from male C57BL/6 mice were subjected to immunohistochemistry and western blot post‐MCAO. Additionally, primary astrocytes were isolated for quantitative real‐time PCR and immunofluorescence assays to evaluate the expression of inflammatory markers glial fibrillary acidic protein (GFAP) and AQP4. AQP4 modulation was achieved using viral knockdown and overexpression methods. Neuronal damage was assessed using flow cytometry and terminal deoxynucleotidyl transferase dUTP nick end labeling (TUNEL) tests in co‐culture studies.

**Results:**

MCAO mice exhibited a significant upregulation in GFAP. This reactive astrogliosis corresponded with an elevation in inflammatory markers. AQP4 expression responded to this inflammatory trend, peaking at 6 h after OGD and returning to baseline levels at 24 and 48 h. Co‐culture experiments revealed that AQP4(+) astrocytes exacerbated injury in OGD‐treated neurons, as evidenced by increased TUNEL positivity and apoptotic events. Conversely, AQP4(−) astrocytes appeared to have a protective effect. Knockdown of AQP4 resulted in reduced post‐OGD inflammatory response, whereas AQP4 overexpression intensified the injury to neurons post‐OGD. In vivo experiments also confirmed that AQP4 inhibitor TGN‐020 reduced and overexpression of AQP4 increased behavioral abnormalities and brain infarcts.

**Conclusion:**

Our findings underscore AQP4's pivotal role in modulating post‐stroke neuroinflammation. Targeting AQP4 may present a novel therapeutic avenue for mitigating ischemia‐induced neuronal damage.

## Graphical abstract

AQP4 is activated during ischemia‐reperfusion, promoting the release of inflammatory factors in astrocytes and accelerating neuronal apoptosis. Inhibition of AQP4 expression supports neuronal survival in the context of MCAO.

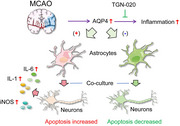

## 1 Introduction

The brain, a highly metabolically active organ, is particularly susceptible to ischemic injuries, leading to a spectrum of disorders collectively termed cerebrovascular diseases (Zhao et al. 2022). One of the principal challenges in understanding these disorders and devising therapeutic interventions is the intricacy of the cellular and molecular responses following ischemic events. A critical player in this milieu, both as a victim and perpetrator of ischemic injury, is the astrocyte (Jurcau and Simion 2021). Expressing the water channel Aquaporin‐4 (AQP4), these cells have gained increasing attention for their roles not only in water homeostasis but also in neuroinflammation following ischemia.

One of the most prevalent cell types in the central nervous system (CNS), astrocytes, are essential for preserving brain homeostasis (Endo et al. 2022). Historically, they were merely seen as supportive cells, filling the gaps between neurons. However, recent decades have witnessed a paradigm shift in understanding their multifaceted contributions to brain physiology and pathology. With their extensive processes that envelope synapses, blood vessels, and other neural cells, astrocytes actively modulate synaptic transmission, regulate cerebral blood flow, maintain ionic balance, and participate in metabolic support of neurons. Recent studies have highlighted the role of astrocytes in neuroinflammation, particularly in the context of ischemic events (Candelario‐Jalil, Dijkhuizen, and Magnus 2022; Di Benedetto et al. 2022). In response to injury, astrocytes undergo various changes, including the expression of specific markers like inducible nitric oxide synthase (iNOS) and arginase 1 (ARG1). These markers are often associated with the polarization of macrophages into M1 (pro‐inflammatory) and M2 (anti‐inflammatory) phenotypes, respectively (Yu et al. 2022; Zhang et al. 2022). While macrophages play a secondary role, their interaction with astrocytes, especially in the context of neuroinflammation, is noteworthy. A critical aspect of astrocytic function is its association with AQP4. This membrane protein, predominantly found on astrocyte foot processes surrounding the blood–brain barrier (BBB), plays a pivotal role in various brain functions (Zhang et al. 2021). Its significance is highlighted by its involvement in cerebral edema, a frequent and often severe outcome of ischemic stroke.

Ischemic events disrupt the delicate balance maintained in the CNS. Previous studies have noted that reduced blood flow leads to oxygen and glucose deprivation, initiating a cascade of events that culminate in cellular damage and death (Kim et al. 2020; Zeng et al. 2022). As neurons are highly sensitive to such changes, they are the first to be affected. Their injury releases a plethora of damage‐associated molecular patterns (DAMPs) and neurotransmitters that can activate microglia and astrocytes (Fan et al. 2020). This activation marks the onset of neuroinflammation, characterized by the release of pro‐inflammatory cytokines, chemokines, and other inflammatory mediators. Astrocytes respond to these changes robustly. Reactive astrogliosis, a hallmark response, involves astrocyte hypertrophy, proliferation, and upregulation of the intermediate filament glial fibrillary acidic protein (GFAP) (J. Wang et al. 2020). While this response aims to limit damage by isolating the injured area, it can also contribute to neuroinflammation, thereby exacerbating neuronal injury.

In the aftermath of a stroke, an immediate response in the brain is the onset of inflammation, a mechanism by which the body tries to combat and contain the damage. In this context, the role of AQP4 may be an influential factor in the neuroinflammatory chain reaction (Chen et al. 2022). Swelling of the astrocytes, in particular, has been linked to increased AQP4 expression, which can further exacerbate the post‐stroke inflammatory response (Datta et al. 2022; H. Wang et al. 2020). Elevated levels of AQP4 can also lead to increased BBB permeability, facilitating the entry of immune cells and inflammatory mediators into the brain tissue. Additionally, interactions between AQP4 and cytokines or chemokines can potentially influence astrocyte activation, migration, and release of pro‐inflammatory substances. Thus, AQP4 is not just a passive participant in post‐stroke neuroinflammation but may actively modulate the dynamics and outcomes of the inflammatory response.

Targeting AQP4 presents a novel avenue for therapeutic interventions in ischemic brain injuries. If AQP4 exacerbates ischemic injury through its involvement in neuroinflammation, modulating its activity or expression could ameliorate outcomes post‐stroke. Conversely, if AQP4 plays a predominantly protective role, strategies to enhance its function might prove beneficial. Determining its exact role is, therefore, of paramount importance. In conclusion, our study explores the mechanisms of AQP4 in the complex events following ischemic brain injury, with a particular focus on the effects on astrocytes. Unraveling the multifaceted roles of AQP4 in ischemia‐induced neuroinflammation could provide insights into potential therapeutic targets, offering hope for improved outcomes in cerebrovascular diseases.

## 2 Materials and Methods

### 2.1 Animals

C57BL/6 male mice (six mice per group), 8–10 weeks of age, were obtained from the Yangzhou University Center for Comparative Medicine (Yangzhou, Jiangsu, China) under license number SCXK (Su) 2022‐0009. Upon arrival, the mice were placed in a regulated setting that maintained a 12‐h light/dark rotation with a steady temperature of 22 ± 2°C and a humidity level set at 50 ± 10%. The mice were given unrestricted access to both water and standard mouse feed. A minimum of 1 week was allowed for the mice to adjust to the lab conditions before initiating any experiments. All mice except the normal group underwent MCAO modeling, the experimental groups were as follows: (1) normal group (*n* = 6) and MCAO group (*n* = 6); (2) MCAO + phosphate‐buffered saline (PBS) group (*n* = 3), MCAO + vector group (*n* = 3), MCAO + TGN‐020 group (*n* = 3), MCAO + ov‐AQP4 group (*n* = 3). Anesthesia was used for all surgical procedures, and the mice were monitored closely during the recovery period. Pain relief was provided as necessary according to the veterinarian's recommendations, in line with the 3Rs principle (replacement, reduction, and refinement) for ethical animal research. The experimental procedures involving these animals received approval from the Animal Ethics Committee of Guangzhou Miles Biosciences (approval number 20230017) and were executed following the guidelines set by the National Institutes of Health for animal care and usage.

### 2.2 Lentivirus Production and Infection

Lentivirus for AQP4 overexpression and knockdown was produced using a third‐generation lentiviral generation system in HEK293 cells from Procell (Wuhan, China). For transfection, 10 µg of lentiviral plasmid was used together with the lentiviral packaging plasmids pMD2.G and psPAX2 lentiviral packaging plasmids in a 4:3:3 ratio. The cells were co‐transfected with the appropriate vector, either the AQP4 cDNA cloned into a pLV‐eGFP‐N‐Puro vector for overexpression (ov‐AQP4) (GenePharma, Suzhou, China) or a short hairpin RNA (shRNA) for AQP4 knockdown (GenePharma), with the sequence of which is: Sense, 5′‐TTAACTAACGTTTGTTTACAGTTCAAGAGACTGTAAACAAACGTTAGTTAA‐3′ and antisense, 5′‐CATTTGTTTGCAATCAATTATTCTCTTGAAATAATTGATTGCAAACAAATG‐3′, integrated into a PLKO.1 vector. As a negative control for both overexpression (ov‐NC) and knockdown (sh‐NC) of AQP4, cells were co‐transfected with an empty pLVX‐IRES‐ZsGreen1 or PLKO.1 vector. Transfections were performed using Lipofectamine 3000 (Invitrogen), according to the manufacturer's instructions, along with the lentivirus packaging plasmids pMD2.G and psPAX2. Lentivirus‐containing supernatants were harvested 48 to 72 h after transfection and centrifuged at 100,000 × *g* for 2 h at 4°C. The lentiviral particles were resuspended in PBS and titrated to 1 × 10^9^ TU/mL by dropwise addition to HEK293 cells, confirmed using fluorescence microscopy (Nikon). For the infection of astrocytes, 20 µL of lentivirus was added to each well of a 6‐well plate containing 1 × 10^5^ astrocytes in DMEM without FBS. To enhance infection efficiency, polystyrene (Solarbio, Beijing, China) was added to a final concentration of 4 µg/mL. After a 6‐h incubation, the medium was replaced with fresh complete culture medium (DMEM + 10% FBS).

### 2.3 Modeling of Middle Cerebral Artery Occlusion (MCAO) in Mice

Mice were anesthetized with 7% chloral hydrate (0.5 mL/100 g), secured to the operating table with medical tape, and their necks shaved. The mice's internal temperature was maintained at 37°C via a feedback‐controlled heating pad from Harvard Apparatus, MA, in the United States. The mouse was given anesthesia before being put in a supine posture with its head held in a stereotaxic frame. The left common carotid artery (CCA), external carotid artery (ECA), and internal carotid artery (ICA) were all visible through a midline neck incision. Following isolation, a temporary ligature was put around the CCA, and the ECA was firmly tied. A monofilament nylon suture (6‐0, Somerville, NJ, USA) was gently introduced into the ICA through an arteriotomy made in the ECA and advanced approximately 9–11 mm from the carotid bifurcation until slight resistance was felt. This position generally corresponds to the origin of the MCA, thus achieving occlusion. The suture was held in place for 60 min to induce ischemia. Mice in the MCAO + TGN‐020 group were administrated intraperitoneally with TGN‐020 (200 mg/kg) or an equal volume of PBS (Gibco, Thermo Fisher Scientific, MA, USA) at this time. After the desired occlusion time, the suture was gently withdrawn to restore blood flow to the MCA territory, simulating the reperfusion phase. The ECA graft was permanently ligated to prevent potential bleeding, and restoration of blood flow to the ICA and CCA was visually verified. The surgical site was then sutured, and the mouse was allowed to recover 2 h from anesthesia under careful observation. Neurological deficits post‐MCAO were scored after 24 h after MCAO modeling using the Longa five‐point scale, where a score of 0 indicates no observable deficit and 4 indicates severe impairment. If the score indicates successful modeling, subsequent TTC (2,3,5‐triphenyltetrazolium chloride) staining of brain sections can be performed. Lentivirus (10 µL) expressing AQP4 (1 × 10^9^ TU/mL) or vector (1 × 10^9^ TU/mL) was slowly injected into the lateral ventricles 24 h before MCAO surgery.

### 2.4 Histological Analysis via Immunohistochemistry (IHC)

Brain sections underwent a 4% PFA fixation and were then set in paraffin. Thin 5 µm sections were crafted and set on glass slides. Post the removal of paraffin and rehydration, citrate buffer was used for antigen recovery. Sections were treated with 5% NGS and then exposed to primary antibodies against GFAP (1:250, ab68428, abcam) in a 4°C setting overnight. After cleansing, sections were exposed to HRP‐linked secondary antibodies and visualized with DAB substrate. The sections were then contrast‐stained with hematoxylin, dried, and sealed.

### 2.5 TTC Staining

TTC staining (Sigma‐Aldrich, MO, USA) was used to delineate the infarcted region after MCAO in our mouse model. Briefly, after behavioral testing, brains were rapidly excised and sectioned into 2‐mm coronal slices using a Leica Microsystems (Wetzlar, Germany) vibratome with cold PBS. These slices were then immersed in a 2% TTC solution and incubated at 37°C in the dark for 15–30 min. Viable tissue, due to mitochondrial activity, converted the TTC solution into a deep red formazan pigment, while infarcted areas remained pale or unstained. After incubation, the sections were rinsed with PBS and fixed in paraformaldehyde for long‐term storage. The stained sections were then photographed, and the infarct volume was determined by taking into account the section thickness and the total brain volume.

### 2.6 Astrocyte Cultures

Astrocytes were primarily cultured from the cortex of newborn rats. After extraction, these cells were placed in T75 flasks pretreated with poly‐L‐lysine. They were then cultured in Dulbecco's modified Eagle's medium (DMEM; Gibco) supplemented with 10% fetal bovine serum (FBS; Invitrogen, Thermo Fisher Scientific) and a 1% mixture of penicillin‐streptomycin (P/S; Gibco). After reaching full growth, the astrocytes were further cultured, and those from the second to fourth cycles were selected for the following tests.

### 2.7 Cell Sorting

The collected astrocytes were resuspended in PBS and incubated with anti‐AQP4 magnetic beads (Dextran‐coated Fe_3_O_4_ nanoparticles, Nanoeast Biotech, Nanjing, China) at the concentration recommended by the manufacturer. This incubation was carried out at 4°C with gentle agitation for 15 min to ensure complete binding of the beads to AQP4(+) cells. The labeled cells were then passed through a MACS separator column (Miltenyi, Germany) placed in a magnetic field. AQP4‐positive cells bound to the beads were retained in the column, while AQP4(−) cells passed through. The column was subsequently removed from the magnetic field, and AQP4(+) cells were eluted by flushing the column with PBS containing 0.5% BSA and 2 mM EDTA. The eluted cells were then stained with a fluorescently labeled anti‐AQP4 antibody and analyzed using a flow cytometer (BD Biosciences).

### 2.8 Mouse Neural Cell Cultures

Neuronal cultures were derived from the cerebral cortices of embryonic mice at E17. Following dissection, the cortices were enzymatically dissociated into a single cell suspension using a solution of 0.25% trypsin‐EDTA. The enzymatic reaction was neutralized using DMEM supplemented with 10% FBS. The cells were plated onto poly‐L‐lysine‐coated dishes in Neurobasal Medium (Gibco) fortified with 2% B27 supplement, 1% P/S, and 2 mM glutamine. Half of the medium was refreshed every 3 days. After 7 days in vitro, when the cultures had reached an adequate level of maturity and cellular connectivity, they were utilized for subsequent experimental assays.

### 2.9 Fluorescent Labeling via Immunofluorescence (IF)

Cells underwent a 15‐min fixation in 4% paraformaldehyde (Beyotime) at ambient temperature, followed by a 10‐min permeation with 0.1% Triton X‐100 (Beyotime). They were then treated with 5% standard goat serum (Burlingame, CA, USA) for an hour. Overnight exposure to primary antibodies at 4°C was followed by a 1‐h exposure to fluorescent secondary antibodies Alexa Fluor 488 Goat‐anti rabbit (ab150077, Abcam) at room temperature. Cell nuclei were marked with DAPI (Thermo Fisher Scientific). Using ProLong Gold antifade solution (Invitrogen), slides were sealed and examined under a fluorescence microscope (Nikon, Tokyo, Japan). Astrocytes marker GFAP primary antibody (1:50) was used to analyze the purity of isolated astrocytes, and neuronal marker β‐III tubulin (1:100, ab18207, Abcam) was used to analyze the isolated neurons.

### 2.10 Co‐Culture of Astrocyte and Neurons

The medium of astrocytes and neuronal cells was mixed in a ratio of 1:1 as a mixed medium for the co‐culture of the two cells. Astrocytes were first deposited at the upper layer of Transwell inserts (Corning) and cultured under DMEM (10% FBS + 1% P/S) for 48 h. Transwell inserts were then placed in culture plates for the neuronal cells and incubated for 10 days, with half of the mixed medium replaced every 3 days.

### 2.11 Oxygen–Glucose Deprivation (OGD) Cell Model

Briefly, after replacing the culture medium with glucose‐deprived DMEM (Gibco), the cells were transferred to an airtight chamber and then flushed with a mixture of 95% N_2_ and 5% CO_2_. After a period of 2 h for neurons or 6 h for astrocytes, the glucose‐deprived medium was replaced with the original culture medium. The cells were then returned to a normal oxygen incubator for reperfusion at intervals of 0, 6, 12, 24, or 48 h.

### 2.12 Protein Analysis via Western Blotting

Astrocyte samples were broken down using RIPA solution (Beyotime, Shanghai, China). Protein levels were gauged with the BCA Protein Measurement Kit (Beyotime). Uniform protein quantities underwent SDS‐PAGE separation and were subsequently moved to PVDF sheets (Millipore, MA, USA). These sheets were treated with 5% skim milk, then exposed to AQP4 (1:1000, ab128906, abcam, Cambridge, UK) and β‐actin (1:1000, ab8226, abcam) primary antibodies in a 4°C environment overnight. After cleansing, the sheets were exposed to HRP‐linked secondary antibodies (PA, USA). Protein patterns were brought to visibility using the ECL Visual System (NJ, USA) and assessed with ImageJ software (MD, USA).

### 2.13 Cell Death Analysis via TUNEL

The TUNEL procedure was executed using the In Situ Cell Death Detection Kit (Beyotime) as per the provided guidelines. In brief, brain slices were permeated with a mix of 0.1% Triton X‐100 and 0.1% sodium citrate for 8 min at 21°C. Post‐PBS washes, slices were treated with the TUNEL reaction solution in a moist chamber at 37°C for an hour. After triple washing with PBS, DAPI was used for a 5‐min nuclei visualization.

### 2.14 Flow Cytometry Examination

Cells were treated with trypsin, cleansed, and resuspended in PBS with 1% FBS. They were then marked with annexin V and propidium iodide (PI) for a 15‐min period in a dark setting. Apoptotic cells were assessed using a flow cytometry device (BD Biosciences, CA, USA). FlowJo software (BD Biosciences) was used for data analysis.

### 2.15 Gene Expression Analysis via RT‐qPCR

RNA from astrocytes and brain samples was isolated using the TRIzol agent (Invitrogen). RNA quality and concentration were verified with a NanoDrop device (Thermo Fisher Scientific). From 1 µg of total RNA, cDNA was synthesized using the SuperScript III Reverse Transcriptase kit (Thermo Fisher Scientific) following the kit's protocol. Quantitative PCR was performed using the SYBR Green Master Mix (Applied Biosystems, CA, USA) on a StepOnePlus Real‐Time PCR System (Applied Biosystems). Specific primers for genes like IL‐1β, IL‐6, IL‐10, iNOS, TNF‐α, CXCL10, AQP4, and the reference gene β‐actin were utilized. The relative gene expression was adjusted to β‐actin and determined using the 2^−ΔΔCT^ approach, and the sequences are shown in Table 1.

**TABLE 1:** Primers used for RT‐qPCR.

Gene	Sequence 5′–3′
IL‐1β Forward	TGCCACCTTTTGACAGTGATG
IL‐1β Reverse	TGATGTGCTGCTGCGAGATT
IL‐6 Forward	CAACGATGATGCACTTGCAGA
IL‐6 Reverse	TGACTCCAGCTTATCTCTTGGT
IL‐10 Forward	AAGGGTTACTTGGGTTGCCA
IL‐10 Reverse	CACCTTGGTCTTGGAGCTTATT
iNOS Forward	CAACAGGGAGAAAGCGCAAAA
iNOS Reverse	CCAGGGATTCTGGAACATTCTGT
TNF‐α Forward	CCCTCACACTCACAAACCAC
TNF‐α Reverse	ACAAGGTACAACCCATCGGC
CXCL10 Forward	GTCTGAGTGGGACTCAAGGGAT
CXCL10 Reverse	TCAACACGTGGGCAGGATAG
ARG1 Forward	GTAGACCCTGGGGAACACTAT
ARG1 Reverse	ATCACCTTGCCAATCCCCAG
AQP4 Forward	TGGGCATCCTGTCACAACAC
AQP4 Reverse	GCAGGAATGTCCACACTTAGACAC

### 2.16 Statistical Analysis

Data were analyzed using GraphPad Prism software (GraphPad Software, CA, USA). Differences between groups were evaluated by one‐way ANOVA followed by Tukey's post hoc test. A *p*‐value < 0.05 was considered statistically significant.

## 3 Results

### 3.1 Astrocyte Reactivity and Inflammatory Markers Altered After MCAO Modeling

**FIGURE 1:**
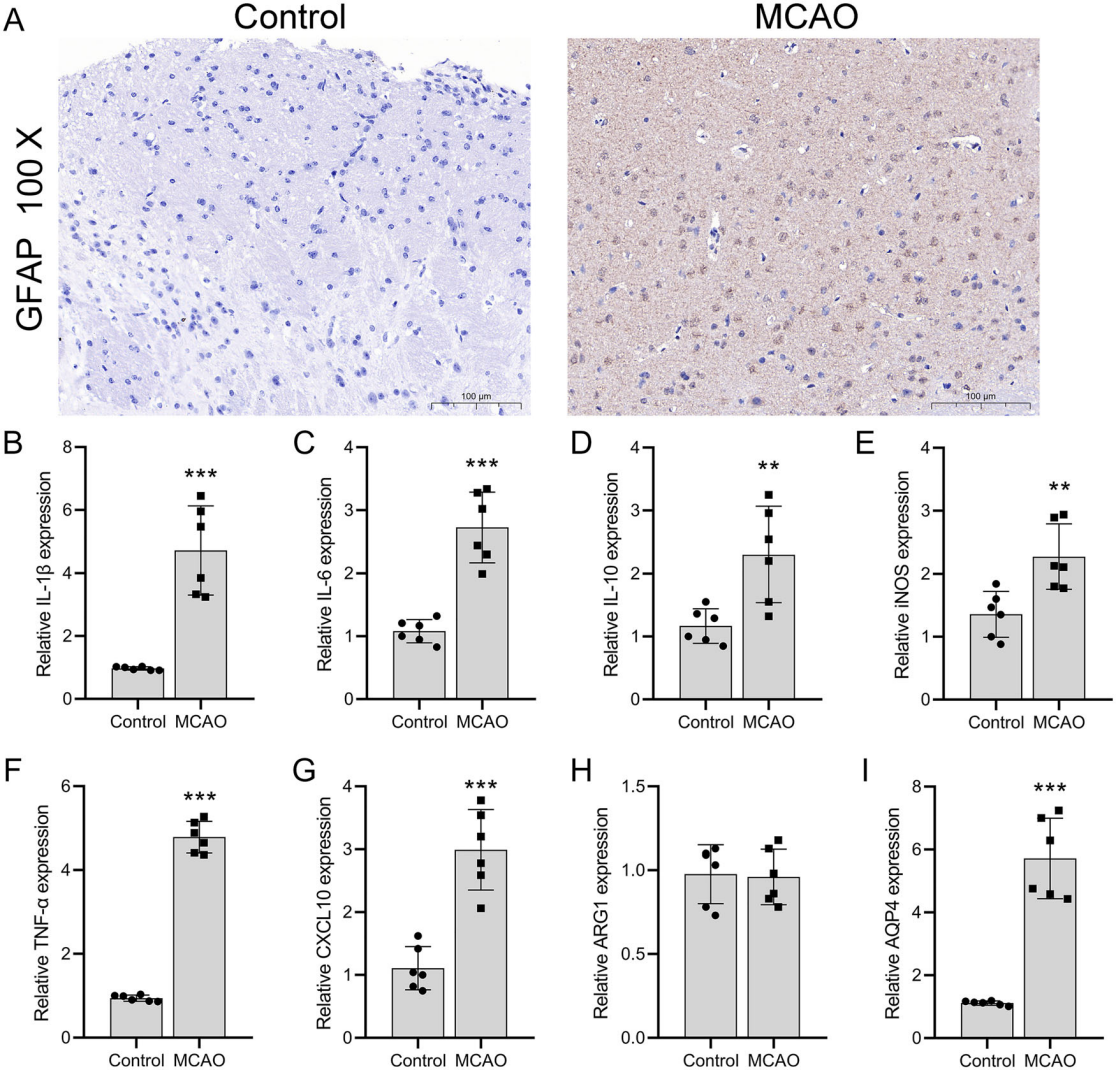
Astrocyte reactivity and inflammatory response after MCAO. (A) IHC analysis of morphological changes in astrocytes post‐MCAO compared to the normal group, highlighting thickened and scar‐like processes. (B–H) RT‐qPCR assessment of quantitative mRNA levels of IL‐1β, IL‐6, IL‐10, iNOS (M1 macrophage marker), TNF‐α, and CXCL10 in brain tissues post‐MCAO compared to normal mice. (I) Immunostaining examination of AQP4 expression levels in brain tissues of MCAO‐induced and normal mice. The data are presented as the mean ± SD. The data are presented as the mean ± SD. *p < 0.05, **p < 0.01, ***p < 0.001, versus control group.

We investigated AQP4's role in astrocytes during post‐stroke inflammation. IHC staining of C57BL/6 male mice brain tissue post‐MCAO model establishment revealed a notable increase in GFAP expression compared to a normal group. This is indicative of reactive astrogliosis, reflecting an increase in astrocyte activation and hypertrophy after injury. Typically, astrocytes in the brain are stellate with predominantly round nuclei. However, post‐MCAO, the processes of astrocytes thickened, and their morphology appeared less ramified, resembling a scar‐like formation (Figure 1A). As anticipated, mRNA levels of IL‐1β (*p* = 0.0003), IL‐6 (*p* = 0.0115), IL‐10 (*p* = 0.0017), M1 macrophage markers (iNOS) (*p* = 0.0001), TNF‐α (*p* = 0.0089), and CXCL10 (*p* = 0.0003) saw significant upregulation (*n* = 3). In contrast, M2 macrophage markers (ARG1) remained unchanged (*n* = 3, *p* = 0.8690) compared to normal mice (Figure 1B–H). AQP4 expression also increased significantly (*p* = 0.0004) in post‐MCAO brain tissues (Figure 1I).

### 3.2 Changes in Astrocyte Morphology and Inflammatory Factors After OGD/R

**FIGURE 2:**
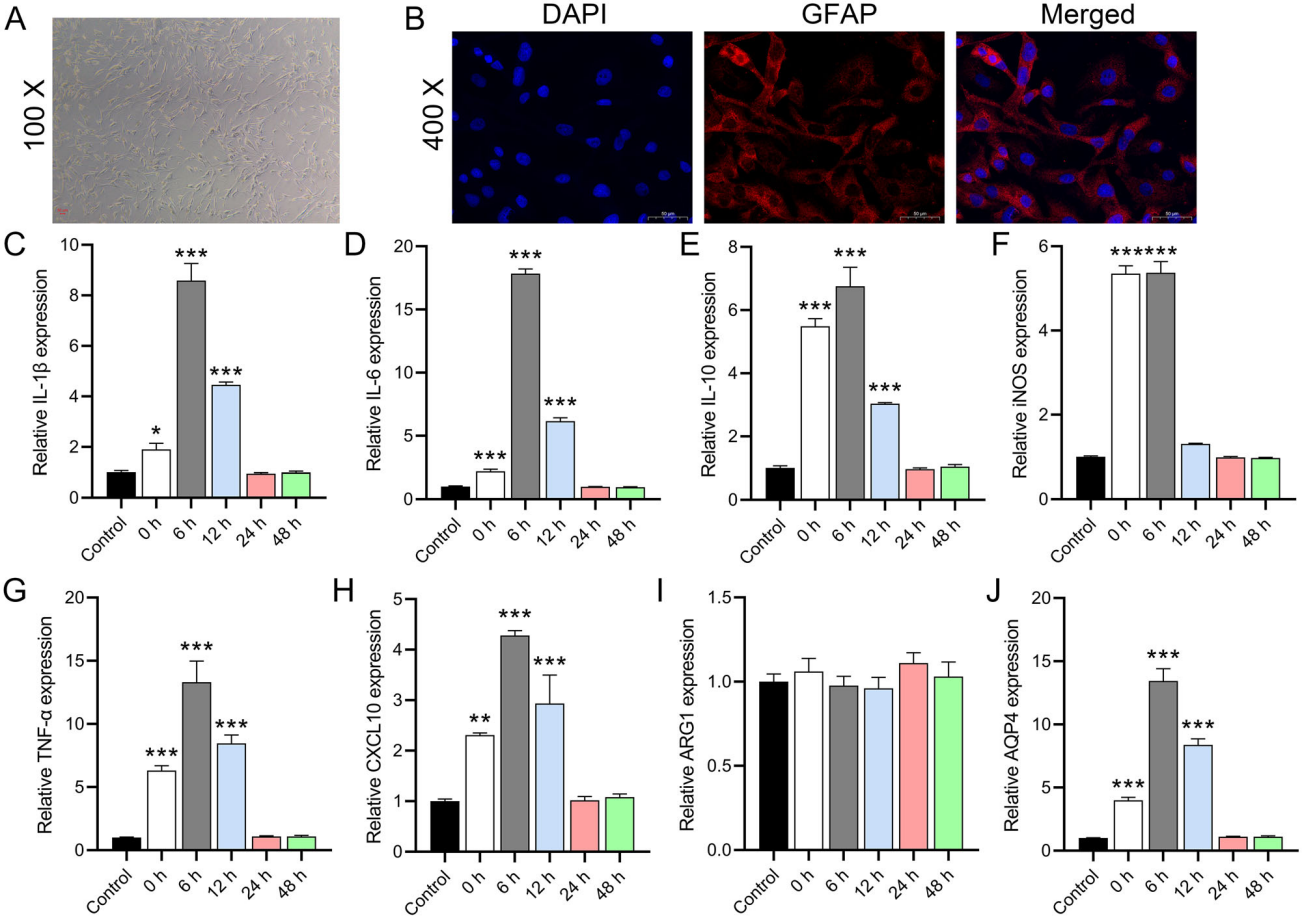
Morphological and inflammatory changes in astrocytes after OGD/R. (A) Microscopic observation of isolated astrocytes showing flattened stellate formation. (B) IF staining evaluation of GFAP expression in astrocytes indicating purity. (C–H) RT‐qPCR measurements of mRNA levels for IL‐1β, IL‐6, IL‐10, iNOS, TNF‐α, and CXCL10 showing a return to baseline levels at 24 and 48 h after OGD/R. (I) RT‐qPCR analysis of ARG1 expression after OGD. (J) RT‐qPCR monitoring of time‐dependent AQP4 expression in primary astrocytes post‐OGD, showing a peak at 6 h. The data are presented as the mean ± SD. *p < 0.05, **p < 0.01, ***p < 0.001, versus control group.

We observed that astrocytes isolated from mouse brain tissue had a flattened stellate morphology and grew in monolayers in close contact with each other (Figure 2A). By IF staining, we found that GFAP was highly expressed in the cells, and the counting results showed that its purity was more than 96.5% (Figure 2B), indicating the presence of a small fraction (4%–5%) of microglial cells. RT‐qPCR analysis showed that the mRNA levels of IL‐1β (0 h, *p* = 0.0258; 6 h, *p* = 0.0001), IL‐6 (0 h, *p* = 0.0001; 6 h, *p *< 0.0001), IL‐10 (0 and 6 h, *p *< 0.0001), iNOS (0 and 6 h, *p *< 0.0001), TNF‐α (0 and 6 h, *p *< 0.0001), and CXCL10 (0 h, *p* = 0.0002; 6 h, *p *< 0.0001) were significantly upregulated at 0 and 6 h, while they returned to baseline levels at 24 and 48 h (Figure 2C–H). ARG1 expression was not significantly changed after OGD (0 h, *p* = 0.8736; 6 h, *p* = 0.9977) (Figure 2I). Due to the proportion of microglia, these results suggest that the increased inflammatory factors were mainly expressed by astrocytes. We further analyzed the AQP4 expression of primary astrocytes at different time points after OGD. The results showed that AQP4 expression peaked at 6 h after OGD and returned to baseline levels at 24 and 48 h (Figure 2J), which was consistent with the trend of inflammatory factors, further suggesting that AQP4 may be a key factor triggering the upregulation of inflammation.

### 3.3 Relationship Between AQP4 Expression and Neuronal Cell Damage Post‐OGD

**FIGURE 3:**
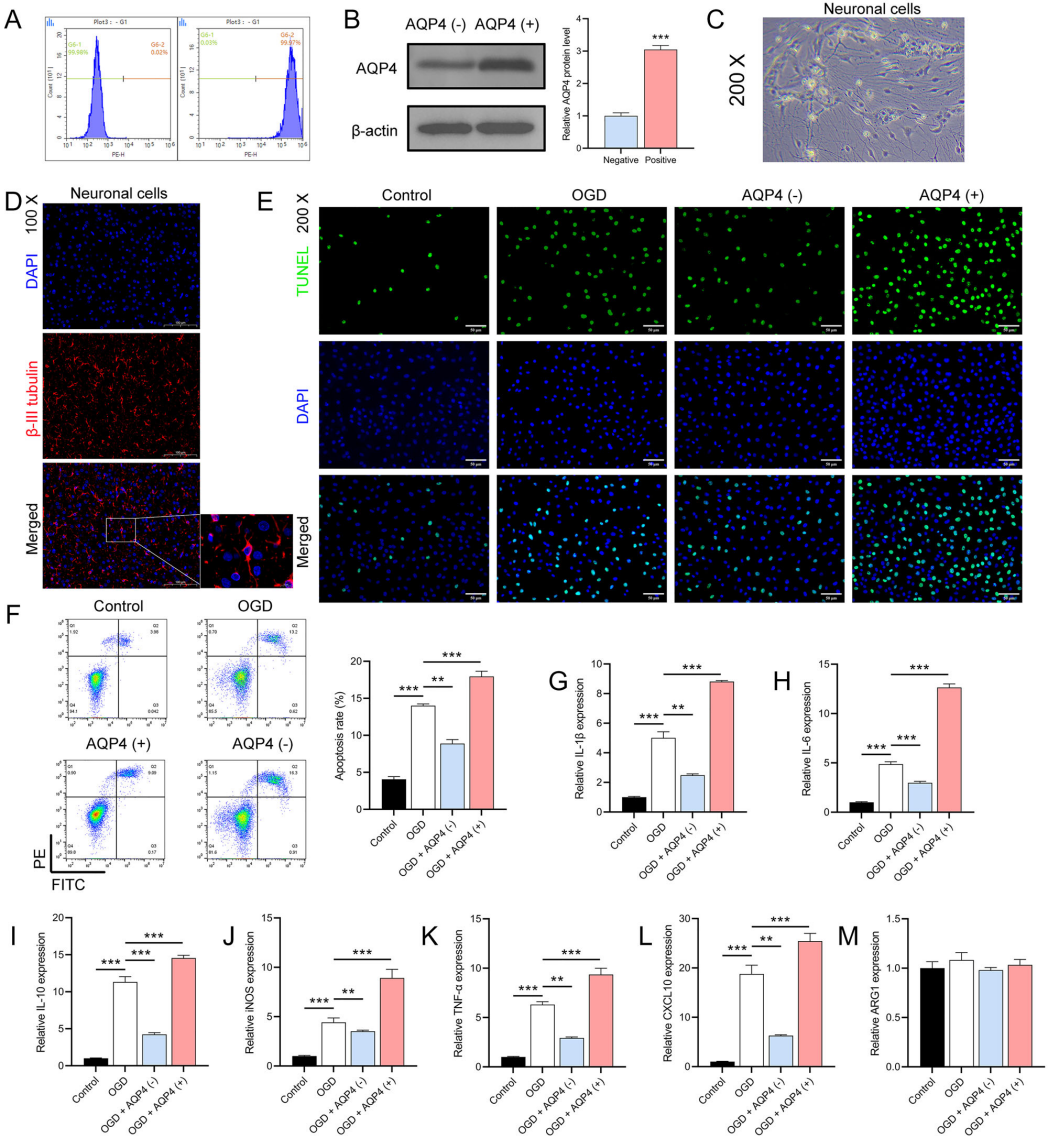
Effect of AQP4 expression on neuronal cell damage after OGD. (A) Flow cytometry‐based purity assessment of AQP4(−) and AQP4(+) cell populations. ***p < 0.001, versus negative group. (B) Western blot validation of increased AQP4 protein levels in AQP4(+) cells. (C) Microscopic depiction of initially isolated neurons showing distinct morphology. (D) IF staining for β‐III tubulin expression, a marker of neurons. (E, F) TUNEL staining and flow cytometry evaluation for apoptosis in neurons co‐cultured with AQP4(−) and AQP4(+) astrocytes. (G–M) RT‐qPCR analysis of inflammatory marker levels in neurons co‐cultured with AQP4(+) astrocytes. The data are presented as the mean ± SD. *p < 0.05, **p < 0.01, ***p < 0.001.

For a deeper exploration of AQP4's function, we employed magnetic beads labeled with AQP4 for cell sorting. Flow cytometry confirmed the high purity of sorted AQP4(−) and AQP4(+) cells at 99.98% and 99.97%, respectively (Figure 3A). A subsequent Western blot corroborated that AQP4 protein levels were significantly higher in AQP4(+) cells (*n* = 3, *p *< 0.0001) (Figure 3B). Freshly isolated neurons exhibited a distinct round or oval shape with clear dendrites and axons (Figure 3C). IF analysis revealed high β‐III tubulin expression, a neuron marker, further establishing the purity of isolated neurons (Figure 3D). Co‐culturing of AQP4(+) and AQP4(−) astrocytes with neuronal cells showed that OGD‐treated neurons paired with AQP4(+) astrocytes had increased TUNEL positivity and apoptosis rates. Conversely, neurons co‐cultured with AQP4(−) astrocytes showed reduced apoptosis (*p *< 0.0001) (Figure 3E,F). RT‐qPCR analysis further emphasized the relationship, showing a significant upregulation of inflammatory markers when OGD‐treated neurons were co‐cultured with AQP4(+) astrocytes (*n* = 3, *p *< 0.0001) (Figure 3G–M). This underscores that AQP4(+) astrocytes may intensify neuronal damage, while AQP4(−) astrocytes may offer a protective effect against such injuries.

### 3.4 AQP4 Knockdown in Astrocytes: Expression and Functional Analysis

**FIGURE 4:**
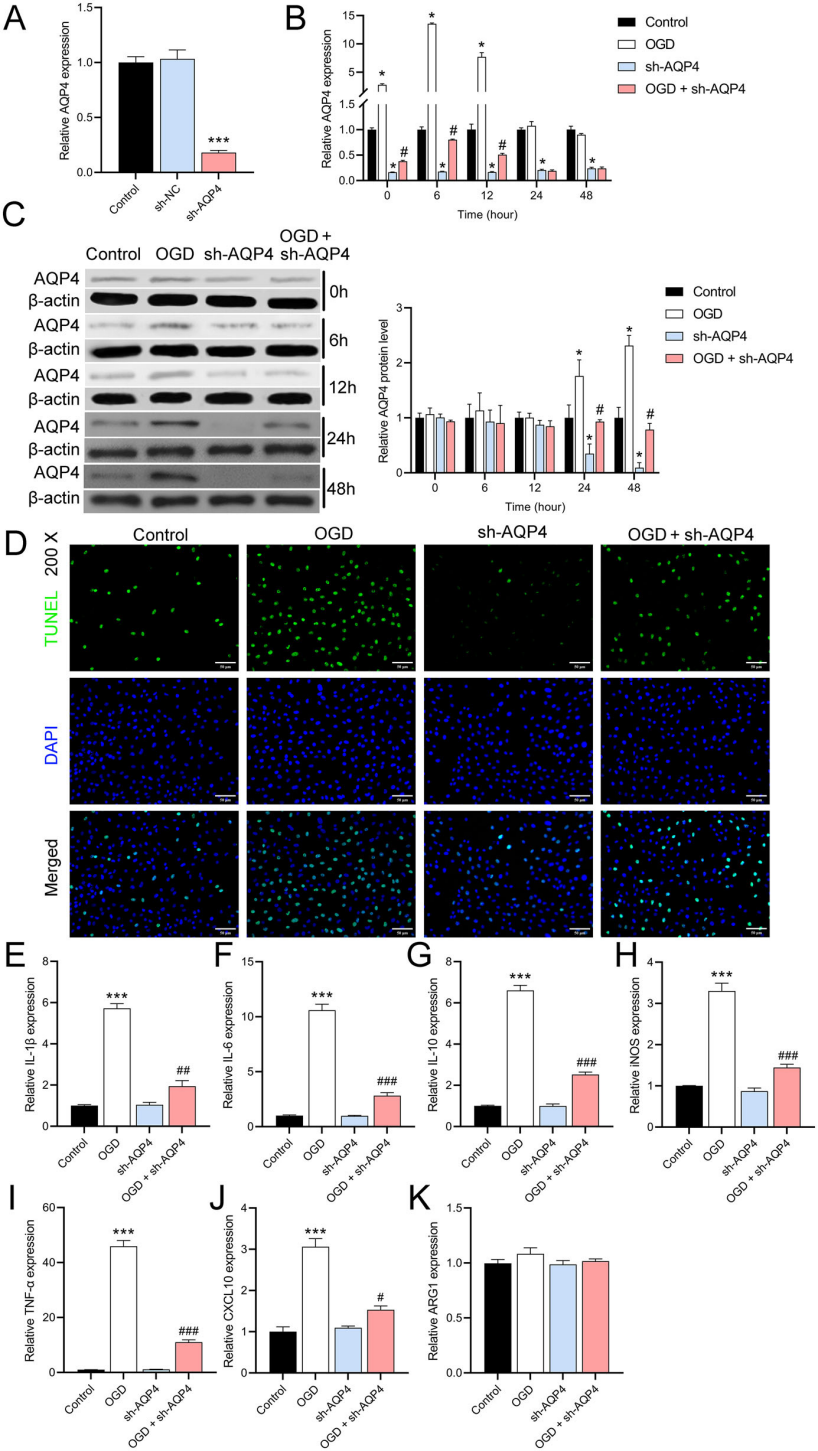
Expression and function of AQP4 knockdown in astrocytes. (A) RT‐qPCR confirmation of decreased AQP4 expression in sh‐AQP4‐infected cells. ***p < 0.001, versus sh‐NC group. (B) RT‐qPCR assessment of time‐dependent AQP4 expression after OGD, with a peak at 6 h. *p < 0.05, versus control group; #*p *< 0.05, versus sh‐AQP4 group. (C) Western blot analysis comparing AQP4 protein levels in astrocytes with AQP4 knockdown and wild‐type cells. *p < 0.05, versus control group; #*p *< 0.05, versus sh‐AQP4 group. (D) TUNEL assay comparison between apoptosis rates in wild‐type astrocytes and AQP4 knockdown cells post‐OGD. (E–K) RT‐qPCR analysis comparing OGD‐mediated inflammation in astrocytes with AQP4 knockdown and wild‐type cells. ***p < 0.001, versus control group; #*p *< 0.05, ##*p *< 0.01, ###*p *< 0.001, versus sh‐AQP4 group. The data are presented as the mean ± SD.

To further explore the role of AQP4, we constructed astrocytes with downregulated AQP4 expression using sh‐AQP4 lentivirus. RT‐qPCR results showed a significant decrease in AQP4 expression in sh‐AQP4‐infected cells compared with normal astrocytes, confirming the efficacy of sh‐AQP4 lentivirus (*n* = 3, *p *< 0.0001) (Figure 4A). RT‐qPCR results showed that AQP4 expression peaked at 6 h after OGD, and AQP4 expression returned to baseline levels at 24 and 48 h. Regardless of whether they received OGD treatment or not, AQP4 expression was consistently lower in cells with knockdown of AQP4 expression (Figure 4B). Western blot results showed that AQP4 protein levels began to be significantly up‐regulated 24 h after OGD compared with normal cells. At the beginning of 24 h, AQP4 protein levels were significantly reduced in AQP4 knockdown cells, confirming that sh‐AQP4 lentivirus began to inhibit AQP4 protein levels at this time point. After OGD, AQP4 protein inhibited by sh‐AQP4 was elevated (Figure 4C). TUNEL results further showed that, compared to wild‐type astrocytes, a large number of TUNEL‐positive cells appeared after OGD, whereas AQP4 knockdown cells exhibited lower rates of TUNEL positivity and reduced the effect of OGD (Figure 4D). RT‐qPCR results showed that knockdown of AQP4 expression reduced OGD‐mediated inflammation (Figure 4E–K).

### 3.5 AQP4 Overexpression's Impact on Neuronal Injury and In Vivo Implications

**FIGURE 5:**
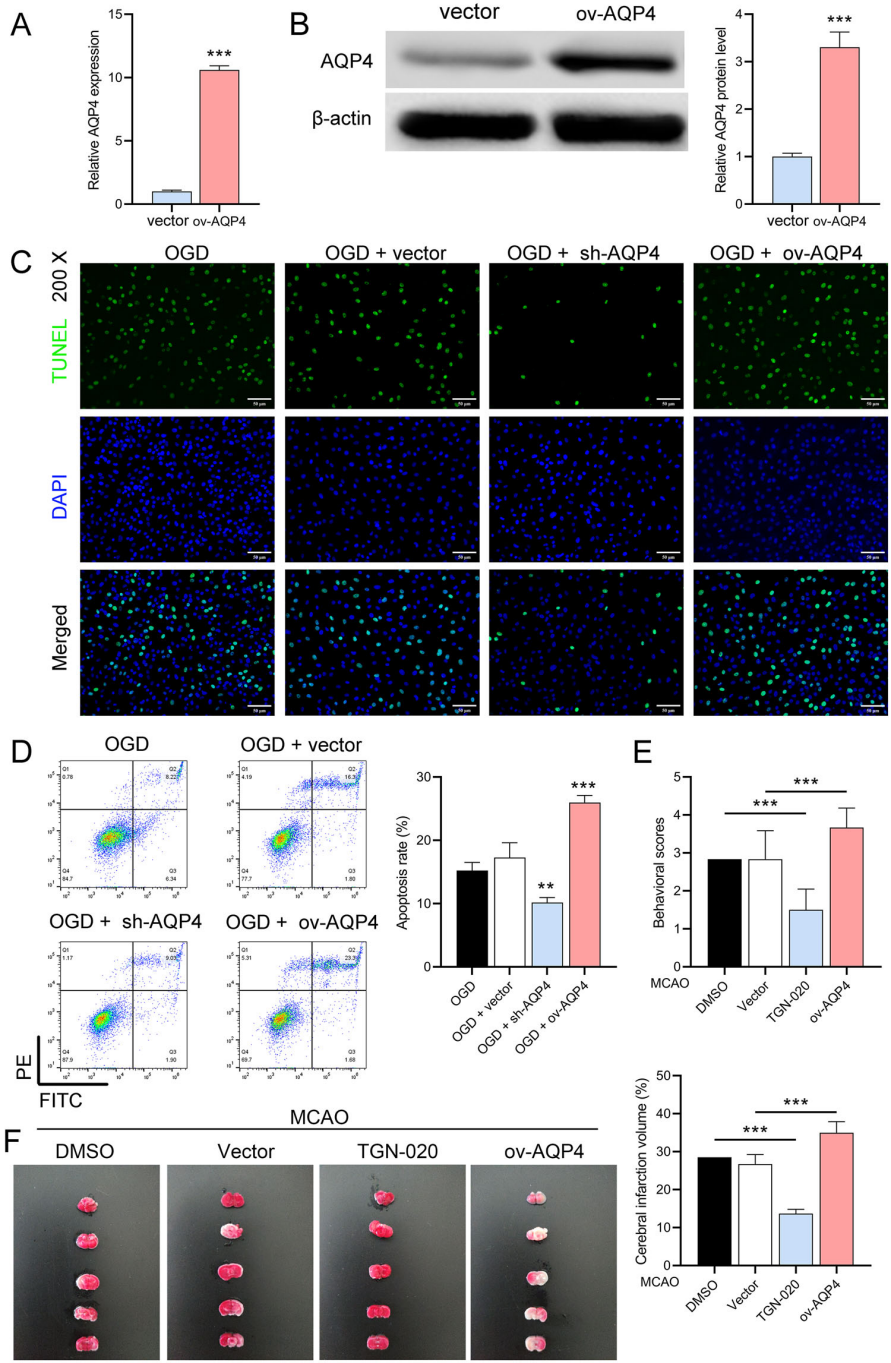
Effect of AQP4 overexpression on neuronal injury. (A,B) RT‐qPCR and Western blot validation of increased AQP4 expression in astrocytes infected with AQP4‐overexpressing lentivirus. ***p < 0.001, versus vector group. (C) TUNEL and flow cytometry assessments of neuronal damage in AQP4‐overexpressing astrocytes post‐OGD. (D) Flow cytometry assessments of neuronal damage in AQP4‐overexpressing astrocytes post‐OGD. (E) Longa five‐point method was used to analyze the effects of AQP4 inhibitor TGN‐020 and AQP4‐overexpressing lentivirus on the neurological deficits after MCAO in mice. (F) TTC staining was used to analyze the effects of AQP4 inhibitor TGN‐020 and AQP4‐overexpressing lentivirus on the ischemic volume of the brain after MCAO in mice, respectively. The data are presented as the mean ± SD. **p < 0.01, ***p < 0.001.

To further investigate the role of AQP4, we used lentivirus overexpressing AQP4 to infect normal astrocytes. We verified the high expression of AQP4 in these cells by RT‐qPCR (*p *< 0.0001) and Western blot (*p* = 0.0003) (Figure 5A,B), and TUNEL and flow cytometry experiments showed that AQP4 overexpression further exacerbated neuronal damage and apoptosis (OGD + sh‐AQP4 vs. OGD + vector, *p* = 0.0018; OGD + sh‐AQP4 vs. OGD + vector, *p* = 0.0005) in astrocytes after OGD (Figure 5C,D). Finally, we performed in vivo experiments on MCAO mice using AQP4 inhibitor TGN‐020 and AQP4 lentivirus overexpression. Longa score and TTC staining results showed that the TGN‐020 was reduced, while AQP4 overexpression exacerbated MCAO‐induced neurological deficits and brain infarcts (Figure 5E,F).

## 4 Discussion

Understanding the intricate molecular pathways involved in brain injury after ischemic stroke is imperative for the development of effective therapeutic strategies. In the present study, we investigated the role of AQP4, a key protein in the maintenance of brain water homeostasis, and its potential impact on post‐stroke neuroinflammation and neuronal apoptosis. Our findings not only elucidate the multifaceted interactions of AQP4 within the CNS but also suggest potential avenues for therapeutic intervention targeting this protein.

In our initial experiments, we established the mouse MCAO model and the astrocyte OGD model. Both models showed upregulation of inflammatory markers compared to the control group, accompanied by increased expression of GFAP in brain tissue. These results confirmed the successful establishment of the MCAO and OGD models (Zhu et al. 2021). Consistent with previous research, we also observed an overexpression of AQP4 in both models, suggesting that AQP4 may play a pivotal role during the ischemia‐reperfusion process (Zheng et al. 2022). Based on the strong observation regarding the modulatory role of AQP4 in astrocytes, we observed a dichotomy. While astrocytes expressing AQP4 exacerbated neuronal damage and inflammation under OGD conditions, astrocytes lacking AQP4 appeared to confer a protective effect on neurons. The expression of iNOS, which is a characteristic marker of M1 macrophages, was increased in AQP4(+) cells, causing significant up‐regulation of inflammatory factors such as IL‐1β, IL‐6, TNF‐α, and CXCL10 expression, suggesting robust activation of astrocytes via the canonical pathway (Chen et al. 2019; S. Wang et al. 2020). After hypoxia, the expression of IL‐10, a marker for M2 macrophages that attempt to suppress excessive inflammation, was elevated, while ARG1 levels remained unchanged, suggesting that the alternative activation pathway does not play a major role here (Liu et al. 2020; Z. Wang et al. 2020). Previous studies have shown that astrocytes and microglia communicate in response to injury (Lyu et al. 2021; Shi et al. 2021); therefore, the interaction between AQP4 and these cells becomes critical in the inflammatory cascade. Our findings on post‐stroke neuroinflammation revealed a positive association between AQP4 expression and inflammatory cytokines.

Another observation in our study was the exacerbation of neuronal apoptosis in an AQP4‐expressing environment. Strong evidence from flow cytometry and TUNEL analysis revealed the pro‐apoptotic nature of AQP4 under ischemic conditions and that knockdown of AQP4 expression had the exact opposite effect. Notably, AQP4 expression peaked at 6 h after OGD and returned to baseline levels at 24 and 48 h. However, AQP4 protein levels began to show significant upregulation at 24 and 48 h after OGD, and AQP4 protein inhibited by sh‐AQP4 was reversed by OGD. This may be due to the fact that protein expression lags behind mRNA expression.

Previous studies have confirmed that the AQP4 inhibitor TGN‐020 reduces astrocyte proliferation by inhibiting AQP4 protein (Li et al. 2024). The Longa five‐point method was used to assess neurological function in the MCAO mouse model and confirmed that GTN‐020 reduced cognitive impairment in MCAO mice. Behavioral deficits were more pronounced in AQP4‐expressing models, suggesting that cellular and molecular changes do indeed translate into functional outcomes. TTC staining results showed that the knockdown of AQP4 expression actually protected brain tissue in MCAO mice and reduced the damage caused by MCAO‐induced ischemia in brain tissue, which was completely opposite to the effect of AQP4.

Although our study has shed light on several aspects of the role of AQP4 after ischemic stroke, it is crucial to acknowledge the potential limitations and confounders. First, the exclusivity of male C57BL/6 mice as our experimental model may limit the generalizability of our findings. Second, the absence of a control group of other variables could lead to confounding factors that could affect the results. Third, this study did not focus on the long‐term effects of AQP4 on MCAO mice, which is lacking in characterizing the chronic phase of neuroinflammation. Fourth, this study did not include clinical analysis and testing, which limits our ability to explore the practical challenges and feasibility of translating the obtained results into clinical applications. These are the focus and direction that we need to pay attention to in the future research.

## 5 Conclusion

AQP4 emerges not merely as a regulator of water homeostasis but as a multifunctional protein intricately involved in post‐stroke neuronal outcomes. Its dual nature—sometimes exacerbating damage, sometimes offering protection—underlines the need for nuanced therapeutic strategies targeting this protein. As the horizon of stroke research expands, delving deeper into the multifaceted roles of proteins like AQP4 will undoubtedly pave the way for innovative treatments.

## Author Contributions

**Xin Xing**: conceptualization, methodology, software, data curation, investigation, validation, formal analysis, visualization, writing–original draft, writing–review and editing. **Shuyan Zhang**: conceptualization, methodology, data curation, funding acquisition, writing–original draft, writing–review and editing, project administration, resources, supervision, formal analysis.

## Ethics Statement

The study animal experiments were approved by the Animal Ethics Committee of Guangzhou Miles Biosciences (approval number 20230017).

### Peer Review

The peer review history for this article is available at https://publons.com/publon/10.1002/brb3.70107.

## Data Availability

The datasets used and analyzed during the current study are available from the corresponding author on reasonable request.
